# Students’ Classroom Silence and Hopelessness: The Impact of Teachers’ Immediacy on Mainstream Education

**DOI:** 10.3389/fpsyg.2021.819821

**Published:** 2022-01-27

**Authors:** Osman Juma, Maysigul Husiyin, Asat Akhat, Imirhamza Habibulla

**Affiliations:** Department of Chinese Language and Literature, Northwest Minzu University, Lanzhou, China

**Keywords:** students’ hopelessness, students’ classroom silence, teacher’s immediacy, silence and hopelessness, teacher instructors

## Abstract

The students’ silence in the classroom has lately become an area of attention of educators and scholars similarly; however, the factors influencing students’ classroom silence are not mainly scrutinized. This construct has been regarded as a problem of the communication between the educator and the learners that not only impact completing the teaching objectives in the classroom but also affect the nurturing of learners’ achievement. In addition, teachers positively have a noteworthy function in learners’ growth and progress and its behavior such as their immediacy remains a significant issue toward stimulating effective educational methods. Whilst teacher immediacy in a classroom setting is important, there is growing awareness about its important effect on learners’ silence and hopelessness. This review tries to provide some considerations about the relationship between teacher immediacy, both verbal and non-verbal, and students’ active silence and hopelessness. Successively, some suggestions are offered to lighten the practice of educators, learners, and teacher instructors.

## Introduction

Learners’ feelings are an integral part of individual well-being and mental health, affecting inspiration, attentiveness, self-adjusting education, and scholastic success (Pekrun et al., 2004). On the one hand, constructive feelings like joy, aspiration, and pride elevate learners’ inspiration to learn, their interest, their utilization of deep learning techniques, and scholastic success (Harley et al., 2019). On the other hand, deconstructive feelings like stress, exasperation, and boredom can jeopardize language education (Dewaele et al., 2018). A person can have a deconstructive mental condition because of such deconstructive emotions. The term hopelessness that should be taken into account in the appraisal of negative constructs is characterized in this regard as all of the deconstructiveness in an individual’s thoughts and feelings regarding the future (OrhanTaşkesen and Uluçay, 2013). When students face failure, they may encounter deconstructive feelings like humiliation and hopelessness, and may not be able to take part in future learning techniques (Zepeda et al., 2020). Since the feelings of success are prominent in education, it is significant to attain information regarding their history that is important for creating an emotionally healthy educational setting that can improve both the mental well-being and performance of learners (Pekrun, 2006).

An intellectual circumstance that drives from deconstructive anticipations regarding the school is known as hopelessness that as it is declared by Pekrun (2006), scholastic type of hopelessness is a deconstructive potential result feeling that arises when people ascribe deconstructive consequences to static causes, like intellectual capacity, and believe that future results will never be better than ones in the past (Pekrun, 2006). Learners facing scholastic hopelessness may thus lose control over anticipated results, avoid extending their commitment to course-relevant activities, leading to reduced performance and learning results (Pekrun et al., 2011). It is an element that reduces inspiration and the determination to live. A person with a weakened determination to live can build circumstances of violence. When the pleasures of life and convictions are diminished, hopelessness is elevated. The level of hopelessness rises directly with the idea that life is pointless and that all ends in death. One who is certain that death is the result is condemned to encounter hopelessness since the quintessence of hopelessness is the conviction of a purposeful life. According to these definitions, hopelessness is elevated when conviction in the future diminishes. Furthermore, when hopelessness is raised, a person might demonstrate violent acts against himself and others (OrhanTaşkesen and Uluçay, 2013). Hopelessness prompts an elevation in negativity about life and the future and diminishment or vanishment of positivity (Lavender and Watkins, 2004).

Alternatively, another issue that is the most explored subject in learning in general and in language learning, in particular, is the problem of learners’ silence in classes. Indeed, silence is regarded as an absence of speech and even the complete absence of hearable verbalization that is extended to involve learners who do not present in a certain topic (Bosacki, 2005). Silence in the class is a broad phenomenon and has turned into an impediment for the association between educators and learners, thereby influencing the accomplishment of instructional objectives in class that is not beneficial for the development of learners’ language skills (Zhouyuan, 2016). Although educators and learners are involved in all activities that consider both past and new information during the educational cycle, most educators face the phenomenon of learners’ silence and this prompts communication errors between educators and learners, and between learners themselves (Zhouyuan, 2016). To this end, as stated by Hanh (2020), educators have made great efforts to involve learners in classroom activities in English as a foreign language (EFL) and to improve the effectiveness of their education, but many learners are again silent and are not interested in taking part in the class. Extensive literature in the study of language teaching has persuasively demonstrated how learners’ silence affects second language acquisition (Wu, 2019; Hanh, 2020; Zhou and Chen, 2020). The most regular one is that these learners are hesitant to take part in class communication; they are not ready to answer; they do not pose inquiries; they are inactive and too reliant on educators (Cheng, 2000) that it seems that this deconstructive relationship with silence may originate from the educators’ anxiety or from their awareness of what defines an educator’s function (Ollin, 2008). In an outdated educational model, educators spend most of their time teaching so they have a highly significant function in-class silence, and all that learners can do is to pay attention quietly; in other words, they do not dare to distract the educator and they do not let ask questions even if some important points are hard to so the primary element behind learners’ silence is related to these conventional educational models, the educator-centric models that impede their involvement and boost their silence understand (Zhouyuan, 2016).

Of course, educators also need to consider learners’ feelings to promote language education and their success (MacIntyre et al., 2019). Emerging from positive psychology (PP), this formulation scrutinize how individuals prosper and grow through life-enhancing strong points and virtuousness (Csikszentmihalyi and Nakamura, 2011; Wang et al., 2021). It was PP that sheds the light to develop the educator-learner association as a humanistic notion and it has been declared that the way learners are preserved in the teaching space influences their learning development (Wang and Guan, 2020; Yan, 2021). Undoubtedly, an important research topic in the field of PP and educational discourse refers to the ways educators could actively help the success of their teaching and learners’ education through discourse (Myers, 2010) because one of the most significant shareholders in all institutional environments are educators and they have a decisive impact on the frequency and nature of success (Pishghadam et al., 2021). In addition to educators’ choices and actions, their behavioral, mental, and didactic characteristics are also at the forefront of education (Derakhshan et al., 2020). All educators want to instruct their learners successfully so they need to know how to develop inspiration in their class and encourage students to actively participate in language learning activities. To achieve this, classroom communication acts may have the configuration of both verbal and non-verbal practices in the class (Tanriverdi Canbaz and Yavuz, 2016).

Originally taken into the mainstream educational communication by Mehrabian (1971), the construct of teacher immediacy refers to the view of physical, expressive, or emotional friendship confirmed through constructive communicative behaviors, and in the educational domain, it is considered as a means to convey the interaction between educators and learners (Sibii, 2010). Communication and association between learners and educators can be enhanced by elevating teacher immediacy. Learners can attain the information they need by communicating more with their educators (Richmond and McCroskey, 2000). Indeed, in both, the fields of communication and education, the study of immediacy holds a long history (Witt et al., 2004, 2010) and it is one of the most effective communication practices of the classroom that has attained more academic interest than most other concepts of educational communication (Zhang et al., 2007; Witt et al., 2010). Likewise, it is one of the commonly considered emotion-raising topics, which leads to the thought of physical, emotive, or emotional closeness that is proved by manners of positive associations (Enskat et al., 2017). Educators with acting abilities can make successful decisions about class practices and functions and can actively contribute to learners’ educational cycle so immediacy can be regarded here as a significant educator practice that has a powerful impact on different dimensions of education and inspiration (Özmen, 2010).

Moreover, immediacy is a significant dimension of social existence in learning and indeed the discerned immediacy of the teacher is related to constructive results like educational effectiveness, course fulfillment, and learners’ educational results (Baker, 2010; Wang and Guan, 2020). In particular, regarding educator-learner communication, the research on the notion of immediacy has shown a connection between educator practices that are immediate or non-immediate and learners’ educational practices, attributes, and results (Fall et al., 2011). According to Swenddal (2011), immediacy is an indispensable asset for language educators in decreasing students’ emotion filters and it is also claimed that it can be changed and enhanced through teaching and training. Immediacy increases the proximity between communicators and reduces physical and/or mental distance (Zhang and Witt, 2016).

Educator immediacy has drawn educators’ interest among the different concepts studied in this area and it alludes to the educator’s verbal and non-verbal practices aimed at lessening the mental and physical distance from learners (Lee, 2019). The former speaks about all the activities which are performed by the teachers. Providing the learners with immediate feedback, carrying on conversations before and after classes while guiding the learners through the conversations are some to mention which consequently result in improving learners’ motivation and their involvement (Cai, 2021). The latter refers to the teachers’ attitude toward their students and how they can minimize their physical and psychological distances with their students and how well they can build up a friendly relationship with them. Teachers’ friendliness, body language, and gestures of friendship, support, and solidarity are some types of non-verbal immediacies that most likely affect the teacher–student relationship (Peng, 2020).

As stated by Kim and Bonk (2010), immediacy incorporates the practices teachers employ to enhance learners’ human association, educator presence, care, and connectivity. These practices assist with developing intimate connections and a sense of intimacy between educators and learners (Gunter, 2007). Learners who are discerned to be more immediate show more amenable educational practices are more willing to participate in class and experience constructive educational results (Witt et al., 2004). Regarding the importance of language educators’ non-verbal immediacy, their non-verbal practices might allow them to bring their students’ deconstructive responses to the utilization of corrective feedback to a minimum (Richmond et al., 2008). The non-verbal immediacy of language educators can advance learners’ participation in the language educational cycle (Witt et al., 2004). Therefore, the non-verbal immediacy practices that a language teacher presents in associations with a student can be regarded as productive (Burroughs, 2007). Learners can be motivated to be more careful throughout the classroom by these productive practices. Therefore, it is worthwhile to scientifically explore how learners determine educators’ non-verbal practices (Özmen, 2010).

In a nutshell, silence can have a positive impact on learners by building space for further awareness and more profound assessments in some scholastic situations on the one hand (Liu, 2005; Tatar, 2005) and on the other hand, some scholars (Nakane, 2002; Tani, 2005) mentioned that silence in language education jeopardizes effective language education when it is defined by the lack of verbal association and reaction from learners. Due to the significance of educator immediacy in all scholastic settings, many investigations have tried to investigate the relation between this relational practice (immediacy) and educator-centric elements like scholastic involvement, commitment, desire to take part in classes, cognitive learning, affective learning, class retention, fulfillment, and state/trait enthusiasm (Derakhshan, 2021; Hussain et al., 2021), based on the researcher’s information, no studies have been carried out to examine the association between this interpersonal behavior, immediacy, and student-related factors such as silence, and hopelessness.

## Review of the Literature

### Teacher Immediacy

The notion of immediacy as a series of practices became apparent in communication studies in the late 1960s. It turns out that individuals are attracted to people and things that they like, appreciate, and have a preference for, and they keep away from what they do not like, what they do not appreciate, and what they do not have a preference for Mehrabian (1971). Immediacy in institutional settings, or the immediacy of educators, lies in a vaster area of educational communication—an interdisciplinary field joining insights from educational psychology, instruction, and communication to explore the communication abilities and capacities utilized by educators and teachers in the cycle of taking part in instructing and education (Rugen, 2018).

Immediacy can be categorized into non-verbal and verbal behaviors and teachers’ immediacy is designated as a group of these two kinds of manners that are prearranged by the educators to make an intellect of intimacy and friendship with the learners (Xie and Derakhshan, 2021). It is known that educator immediacy affects learners’ emotional, behavioral, and intellectual educational domains, and elevates their inspiration (Wendt and Courduff, 2018). Educators can capture students’ motivation and preserve their enthusiasm for learning if the educator employs immediacy (Hsu, 2010). Verbal immediacy practices are directly utilized by educators and are significant in building satisfaction or dissatisfaction in learners toward educators. They are the fastest-growing ones between educators and learners (Marcia et al., 2016). These practices generally include humor, calling by names, offering individual examples, and sharing of experiences, motivating learners to associate with educators inside and outside (Witt et al., 2004). Educators’ verbal immediacy alludes to oral messages that demonstrate compassion, openness, friendliness, prize, acclaim, inclusiveness, comedy, individual information, and, above all, the willingness to involve learners in communication (Ballester, 2015). However, non-verbal educator immediacy involves non-oral attitudes that advance “physical and emotional” intimacy and draws learners’ attention to the teacher, the research, and the class material (Richmond and McCroskey, 2000).

Practices that improve mental proximity between communicators are known as non-verbal immediacy. It is incorporated into the strengthening framework that underlies the theory of relational attraction. This concept primarily applies to a person’s emotional discernment of another (Allen et al., 2006; Wang and Guan, 2020). Reinforcement is a general subject of relational attraction theories. The enhancement theory, as one of the fundamental concepts of psychology, contends that actions with desirable results incline being reproduced. This principle, which applies to relational connections, proposes that when a person communicates with another, he/she should want to continue communicating with that person as soon as he/she finds something of value. In the educational setting, non-verbal actions that educators use when communicating with learners, if regarded as holding value, can help advance learners’ participation in the class (Witt et al., 2004). Non-verbal immediacy practices are generally characterized as the indirect utilization of behavioral signs inducing immediacy and they include body language, gesticulation, facial expressions, body motions, and outfit messages (Wendt and Courduff, 2018). Non-verbal educator immediacy practices are characterized by nodding constructively, physical distance, the effect of voice articulation, grinning, eye-to-eye contact, comfortable body position, utilizing emotions, and facial expressions when talking (Marcia et al., 2016). Non-verbal immediacy highlights the multifaceted characteristics of associations and the deliberation of all social signs in the setting of one another. This standard offers a “social” characteristic that can be corresponded against a result, like learning, and can be contrasted against another group of practices defined in the same way (Witt et al., 2004).

### Hopelessness

Scholastic hopelessness is a deconstructive result consequence. People who experience hopelessness ascribe unwanted consequences they experience in life to static factors such as intellectual capacity and have low expectations for future results based on their prior experiences (Pekrun, 2006). Learners who are facing scholastic hopelessness do not want to focus on activities that hinder their accumulation and acquisition of knowledge because they cannot control future results, and this ultimately prompts poor scholastic performance (Pekrun et al., 2011). Concisely, hopelessness refers to a person who feels negative about the future, causing depression and it is deemed as one of the most reliable and vigorous issues of anxiety and it refers to the destructive prospects about the future in coping with their difficulties (O’connor et al., 2000). In the educational context, it is maintained that hopelessness is predominantly destructive to learners’ enthusiasm (Pekrun et al., 2004). Likewise, it has been revealed that despair and unhappiness have a harmful result on working memory presentation (Hubbard et al., 2016), and consequently, despair can be thought parallel to embarrassment and apprehension in its consequences on L2 presentation in this realm. Based on the review of literature, not only hopefulness but also hopelessness is the practicable reflection of an individual’s views to achieve his/her real purposes in the future. While there is anticipation in hopefulness where the tactics and policies to achieve the objective will be accomplished, an assumption of failure is existent in hopelessness and these two prospects may be diverse from individual to individual or circumstances to circumstances based on the way, the projected condition will take place (Karakus, 2018).

### Silence

Silence is reflected to be a subtle notion that cannot be consensually elaborated. Even though one’s perception may propose that silence is a formal negated of communication, scholars have claimed that it is vague and burdened with numerous communicative significances (Liu, 2005). Through academic viewpoints, silence is inevitably secured with feeling, as non-verbal communication tends to be more distinctive than verbal communication, which is intellectual (Gregersen, 2007). In general, silence should be placed amongst non-verbal communication, while it is verbal in positive precise communicative occurrence. Silence may be referred to as one of the procedures a speech act may take when it has a planned communicative utility and consequently silence works are part of the voiced program (Zhouyuan, 2016). Bosacki (2005) declared various explanations of silence like this: a sign of elimination; the distress of societal assessment; a sense of invisibility; expressing social indifference; strong academic commitment. The description of silence specifies that silence both comprises plentiful common content, and takes some particular cultural suggestions and societal attitude with the national features. In addition, silence reflects the noiseless non-verbal communicative behavior that students act in the teaching process in the classroom (Bosacki, 2005). Moreover, non-verbal communication is believed to redirect the speakers’ emotions more precisely than verbal communication (Gregersen, 2007).

Based on the modification of the efficiency of classroom silence, it is categorized into two types, namely the positive silence and negative silence. While the former refers to the state that learners are pondering after educators’ inquiring, the latter talks about the emotive aspects that learners show no interest, no care, no involvement in the learning subjects (Teng, 2009). Moreover, Teng (2009) claimed that learners’ silence in the process of learning refers to a type of emotional state and manners a learner offers in the level of thought, emotion, and achievement that is unrelated and indifferent to the teaching matters. Silence in the classroom can be in the practice of non-contribution inclines to be regarded destructively, often bringing about emotions among classroom contributors. The silence and successive negative classroom atmosphere often affect the emotive and academic performances of the learners and educator (Smith and King, 2018). Alemi and Pashmforoosh (2012) declared that more caring educators inspire students to be more communicative in the educational setting and accordingly, the way educators act and perform in this domain can influence the teaching and learning procedure, and it can be an issue impacting learners’ silence in the classroom. Silences of the learners and their feeling have multifaceted indices in the classroom that cannot be simply anticipated or alleviated even by the dominant educational approaches (Smith and King, 2018). A classroom’s emotive setting is manifestly integrated into the process that educators’ and learners’ feelings are observed. Educators regularly misunderstand learners’ emotive manners (Chang and Davis, 2009); particularly non-verbal communication, since there are no aural signals to understand and educators depend on accurately identifying non-verbal emotive signs.

## Implications and Future Directions

The present review provides many suggestions and contributions. Immediacy, verbal/non-verbal, is one of the practices teachers should have in class and it can have a significant impact on learners’ characteristics. Based on the review of previous studies, enhancing educators’ immediacy developing practices will promote learners’ education, stress tolerance, self-realization, and self-confidence, thereby reducing their level of hopelessness. Likewise, this review helps managers and officials select the best educators for their school or faculty. In addition to the teacher’s knowledge, other important educator attributes like class management skills and the capability of building immediate connections with learners must also be regarded as effective elements. Educators can give their best to optimize their utilization of immediacy during instruction by grinning, having eye contact with all learners, motioning, utilizing voice swings, moving in class, and possessing an open body gesture.

Furthermore, given the connection between feelings and discerned education, educators need to take into consideration how their behavior affects learners’ feelings. Teachers can advance pride and aspiration by recognizing learners’ work and offering constructive feedback. Moreover, teachers, who implement both types of immediacy, that is to say, verbal and non-verbal signs properly, being accessible to learners not only in the classroom but also out of the classroom and smiling, assist their students to be conscious of their aptitudes, capabilities and accordingly grasp their perspective that in this way, and due to this closeness, their levels of silences decreases and they become hopeful. Immediacy practice can be argued to prompt learners to better attain information, be more creative, develop, and increase their skills.

It is advisable for teaching policymakers and officials to focus more on offering courses to enhance educators’ immediacy due to the significance of verbal/non-verbal immediacy in education and its specific impact on learners’ education and feelings. Immediacy enhancement courses offer educators a great chance to learn more about the significance of educational communication in the class and to help develop both verbal and non-verbal practices.

English language organizations and school officials must ensure that educators with high verbal/non-verbal immediacy are selected to assist learners with burnout, low self-confidence, and other elements. Educator trainers are the other shareholders who will make use of this research by fostering the knowledge and immediacy execution in novice educators. They can provide classes, conferences, webinars, and other training programs where the educational, mental, interpersonal, and emotional dimensions of the classroom are instructed as well. In addition, Educators communicate continuously with learners through body language, glimpses, motions, and facial expressions. For this reason, teachers must be careful and consistent in teaching their learners constructive emotions and expressions. Educator trainers can help them by modeling practices that will allow them to acquire the abilities of verbal and non-verbal immediacy. This can be done by praising learners’ endeavors in the class, motivating them to speak, and openly and actively communicating with them outside the class via email, and so on (Velez and Cano, 2008).

## Author Contributions

All authors listed have made a substantial, direct, and intellectual contribution to the work, and approved it for publication.

## Conflict of Interest

The authors declare that the research was conducted in the absence of any commercial or financial relationships that could be construed as a potential conflict of interest.

## Publisher’s Note

All claims expressed in this article are solely those of the authors and do not necessarily represent those of their affiliated organizations, or those of the publisher, the editors and the reviewers. Any product that may be evaluated in this article, or claim that may be made by its manufacturer, is not guaranteed or endorsed by the publisher.
